# The Ross Operation in Children—What Is the Long-Term Fate of the Autograft?

**DOI:** 10.1093/icvts/ivag176

**Published:** 2026-06-29

**Authors:** Nabil Hussein, Tristan Ramcharan, Joseph George, Natasha E Khan, Timothy J Jones, Phil Botha

**Affiliations:** Paediatric Cardiac Surgery, Birmingham Children’s Hospital, Birmingham, B4 6NH, United Kingdom; Paediatric Cardiology, Birmingham Children’s Hospital, Birmingham, B4 6NH, United Kingdom; Paediatric Cardiac Surgery, Birmingham Children’s Hospital, Birmingham, B4 6NH, United Kingdom; Paediatric Cardiac Surgery, Birmingham Children’s Hospital, Birmingham, B4 6NH, United Kingdom; Paediatric Cardiac Surgery, Birmingham Children’s Hospital, Birmingham, B4 6NH, United Kingdom; Paediatric Cardiac Surgery, Birmingham Children’s Hospital, Birmingham, B4 6NH, United Kingdom

**Keywords:** Ross operation, autograft, aortic valve, Shone complex, congenital heart disease

## Abstract

**Objectives:**

The Ross operation achieves excellent outcomes in children and adolescents, but the influence of age at surgery and underlying pathology on long-term results remains uncertain. Evidence on autograft dilatation necessitating reintervention is also limited. This study aimed to identify patient groups best served from the Ross procedure and to evaluate long-term autograft performance.

**Methods:**

All consecutive patients <18 years undergoing a Ross or Ross-Konno procedure between 1991 and 2024 at a single centre were analysed. Demographic, operative, and follow-up data were collected. Patients were stratified into age groups (neonate, infant, child, adolescent) and diagnostic groups (isolated aortic valve disease, aortic stenosis with subaortic stenosis, Shone’s complex, and complex congenital heart disease [CHD]). Outcomes included overall survival, freedom from autograft and right ventricle-pulmonary artery (RV-PA) conduit reintervention, and severe autograft dilatation (>50 mm or >25 mm/m^2^).

**Results:**

A total of 226 patients underwent surgery at a median age of 8.5 years; 67% were male. Median follow-up was 9.5 years (IQR 12.7). Operative mortality was 2% and overall mortality 5%. Survival exceeded 90% at 30 years in all groups except neonates (60% at 5 years). After adjustment for age, Shone’s complex and complex CHD were associated with an 11-fold and 8.8-fold increased hazard of death, respectively (*P* < .03). Freedom from autograft reintervention was 82% and from RV-PA conduit reintervention was 66% at 25 years. Freedom from severe autograft dilatation was 71% at 20 years by absolute dimension and 54% when indexed to BSA. Greater than mild aortic regurgitation at discharge conferred a 17-fold risk of autograft intervention (*P* < .001).

**Conclusions:**

The Ross procedure provides excellent long-term survival and valve durability in children and adolescents, particularly those with isolated aortic valve disease. Autograft dilatation and conduit reintervention remain important late risks, reinforcing the need for lifelong surveillance in specialised centres.

## Graphical abstract

**Figure:**
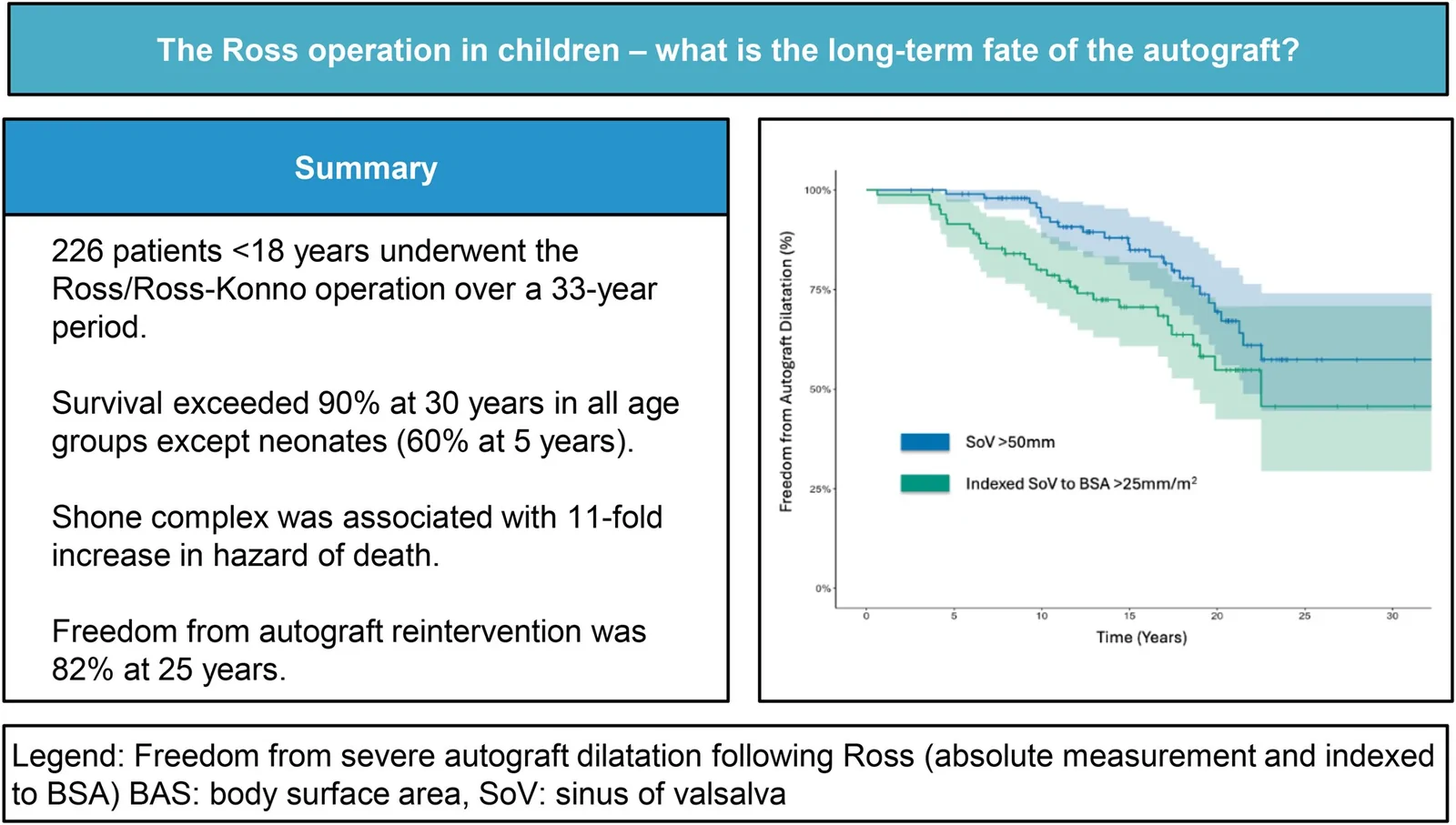

## INTRODUCTION

The Ross operation remains the preferred method for aortic valve replacement in patients undergoing surgery during somatic growth. This involves transposing the patient’s native pulmonary valve into the aortic position and re-establishing the right ventricle to pulmonary artery (RV-PA) continuity with a conduit.^1^ In children, this technique is particularly advantageous due to the growth potential of the autograft, avoidance of anticoagulation, and limitations of small prosthetic valves. While survival outcomes in children are excellent, concerns remain regarding autograft durability and the long-term risk of reintervention.^2–4^ Although recent multicentre studies have been published, paediatric Ross studies are limited to single-centre studies or focused on specific age categories.^4^^,^^5^ Neonates and infants have the highest operative mortality and worst long-term survival. This likely reflects greater complexity, as these patients often present in a decompensated state with few alternatives, or develop obstruction of multiple left heart structures.^6^ Few studies have analysed the impact of age at intervention and disease pathology on long-term outcomes following the Ross procedure and data on autograft dilatation requiring reintervention is limited. This study aimed to identify the patients best served by the Ross operation and evaluate long-term autograft performance.

## METHODS

### Patients and methods

This retrospective, single-centre cohort study included all consecutive patients under the age of 18 years of age who underwent the Ross operation between April 1991 and December 2024. Operations were performed by 6 surgeons over the 33-year period. Data collected from hospital records included: baseline demographics, the primary congenital lesion, category of aortic valve disease, operation performed, and post-operative course (Table 1). Procedures performed prior to the Ross operation were recorded and analysed based on the original valve pathology (Table 2). The main indication for surgery was classified as stenosis, regurgitation, or mixed aortic valve disease.

**Table 1.** Baseline Characteristics of Patients Undergoing the Ross Operation

Age (median ± IQR)	8.5 ± 9.9	
Weight (median ± IQR)	26 ± 32	
Male	151	67%
Mechanism of pathology at Ross		
Aortic stenosis (native)	52	23%
Aortic stenosis (postintervention)	2	1%
Aortic regurgitation (native)	20	9%
Aortic regurgitation (postintervention)	40	17%
Mixed disease (native)	30	13%
Mixed disease (postintervention)	81	36%
Other	1	0%
Primary lesion		
Congenital stenosis	163	72%
Dysplastic aortic valve	24	11%
Shone’s	21	9%
Endocarditis	4	2%
Homograft failure	2	1%
Prosthetic valve dysfunction	2	1%
Other	10	5%
Aortic valve morphology		
Tricuspid	60	27%
Bicuspid	143	63%
Unicuspid	3	1%
Indeterminate	19	9%
Other	1	0%
Category of aortic valve disease		
Isolated aortic valve	135	60%
Aortic stenosis + subaortic stenosis only	58	26%
Shone complex^a^	21	9%
Complex CHD includes: DORV, heterotaxy, hypoplastic arch + VSD or IAA)	12	5%

a Shone complex defined as aortic stenosis with at least 2 of the following: supravalvular mitral ring, parachute mitral valve, subaortic stenosis, or coarctation of the aorta.

**Table 2.** Pre-Ross Operation Interventions (Surgical and Catheter)

Any intervention (surgical + catheter based)	169	75%
Surgical interventions	129	56%
Number of surgical interventions		
1	92	72%
2	29	23%
3	5	4%
4	2	2%
5	1	1%
Type of surgical intervention		
Surgical valvotomy ± subvalvar resection	83	
Subvalvar resection only	21	
Arch repair ± valvotomy ± subvalvar resection	13	
Aortic valve repair ± replacement	14	
Coarctation of aorta repair	14	
Mitral valve replacement	2	
Other	13	
Catheter intervention (balloon dilatation of aortic valve)	72	
Time from first intervention to Ross in years (median ± IQR)	6 ± 8	

Patients were categorised into age groups: neonates (≤28 days), infants (29 days-1 year), children (1-12 years), and adolescents (12-<18 years) as well as diagnostic groups: (1) isolated aortic valve disease, (2) aortic stenosis and subaortic stenosis only, (3) Shone complex (aortic stenosis with at least 2 of the following: subaortic stenosis, supravalvular mitral ring, parachute mitral valve, coarctation of aorta); or (4) complex CHD (double outlet right ventricle, heterotaxy, hypoplastic aortic arch + ventricular septal defect, or interrupted aortic arch).

The Ross and Ross-Konno operations were performed using the freestanding aortic root replacement technique for autograft implantation using 2 layers of continuous polypropylene suture lines to implant the root. In cases with LVOTO, the outflow tract was enlarged using the modified Konno technique.^7^ Cryopreserved pulmonary homografts were used for the RV-PA conduit whenever available, alternatives included aortic homografts or bovine jugular vein conduits.

Follow-up data included clinical outcomes and serial imaging (echocardiogram and magnetic resonance [MRI]) from hospital discharge to determine autograft and RV-PA conduit function. Primary end-points included overall survival, freedom from aortic valve reintervention, significant aortic root dilatation, and RV-PA conduit reintervention. Operative mortality was defined as 30-day or in-hospital mortality. Major morbidity included: cardiac arrest, low cardiac output syndrome, requirement for extracorporeal membrane oxygenation (ECMO), neurological injury, permanent pacemaker, diaphragmatic palsy, mediastinitis, renal failure, and need for urgent reoperation. Aortic root measurements were determined by serial MRI including absolute dimensions of the sinuses of Valsalva and dimensions indexed to body surface area (BSA). Defined cut offs for severe dilatation were >55 mm (absolute) or >25 mm/m^2^ (indexed to body surface area).^8^ MRI surveillance was not performed routinely but was guided by clinical indication, typically in patients with evidence of autograft dilatation or aortic regurgitation on echocardiography, consistent with previously reported practice.^9^

The study was registered with the institutions clinical audit department; however in accordance with UK NHS National Research Ethics Service guidance, neither individual informed consent nor formal research ethics committee review was required as the study was undertaken by the direct clinical team using information previously collected during routine patient care.

### Statistical analysis

The descriptive statistics are presented as median and interquartile range for continuous variables. Time-to-event outcomes were analysed using Kaplan-Meier survival analysis. Survival curves were stratified by diagnostic group and compared using log-rank test. Multivariable Cox proportional hazard models were constructed to assess factors associated with mortality, reintervention, and autograft dilatation. Proportional hazards assumptions were verified using Schoenfeld residuals. Variables for inclusion were selected based on clinical relevance and prior literature. Autograft root dimensions were visualised over time using Sankey diagrams to demonstrate progression. Statistical analysis was performed using R version 4.4.1 (R foundation for Statistical Computing, Vienne, Austria).

## RESULTS

Over the 33-year period, 226 patients underwent a Ross or Ross-Konno operation at a median age of 8.5 ± 9.9 years and median weight of 26 ± 32 kg. One hundred and fifty-one (67%) patients were male. There were 5 (2%) neonates, 31 (14%) infants, 125 (55%) children, and 65 (29%) adolescents. Mixed aortic valve disease was the most common mechanism of pathology at Ross (111, 49%). Most patients had isolated aortic valve disease (135, 60%), followed by aortic stenosis + subaortic stenosis (58, 26%), Shone complex (21, 9%), and complex CHD (12, 5%).

A total of 156 (69%) underwent a Ross, 53 (23%) a Ross-Konno, and 17 (8%) a Ross + non-Konno subvalvar resection. The sinotubular junction (STJ) was reinforced in 9 (4%) patients with dilation of the ascending aorta or requiring concomitant ascending aortic replacement using a short Dacron tube. Thirty-two (14%) underwent an additional procedure at the time of Ross, with mitral valve repair being the most common (Table 3). Pulmonary homografts were used for the RV-PA conduit in 191 (85%) followed by aortic homograft 20 (9%) and bovine jugular vein in 15 (6%). Median cardiopulmonary and cross-clamp times were 146 ± 40 and 108 ± 33 min, respectively.

**Table 3.** Operative Details at Ross

Total number of Ross operations	226	
Neonate (<28 days)	5	2%
Infant(29 days-1 yr)	31	14%
Child(1-12 yrs)	125	55%
Adolescent(12-<18yrs)	65	29%
Type of operation		
Ross	156	69%
Ross Konno	53	23%
Ross+subAS resection(not Konno)	17	8%
STJ reinforcement^a^	9	4%
Additional procedure at Ross	32	14%
Arch repair ± other	5	2%
Ascending aorta replacement	6	3%
Reinforcement of STJ with Cuff	3	1%
MV repair	9	4%
MV replacement	2	1%
Other	7	3%
Conduit used		
Pulmonary homograft	191	85%
Aortic homograft	20	9%
Contegra	14	6%
Tissue med	1	0%
Cardiopulmonary bypass time in min (median ± IQR)	146	40
Cross clamp time in min (median ± IQR)	108	33

a STJ reinforcement—ascending aorta replacement or STJ cuff.

### Pre-Ross intervention

Overall, 169 (75%) patients had an intervention on the aortic valve prior to the Ross operation, with 129 (56%) having had a surgical intervention and 72 (32%) a balloon dilatation. The median age of pre-Ross intervention was 39 days with surgical valvotomy ± subvalvar resection being the most common intervention in the cohort. The median time from first intervention to Ross was 6 ± 8 years. Of the 129 patients who had pre-Ross surgical intervention, 80 patients had only surgical aortic valve interventions (surgical valvotomy with or without valve repair and/or subaortic resection), the median time to Ross was 5.9 ± 9 years. Among the 72 patients who underwent balloon valvotomy, 34 had catheter-based interventions only, with a median interval to Ross of 4.5 ± 8.7 years. Thirty-eight patients underwent both surgical intervention and balloon valvotomy prior to the Ross procedure, with a median interval from first procedure to Ross of 6.4 ± 9.1 years.

### Survival

**Figure 1.**
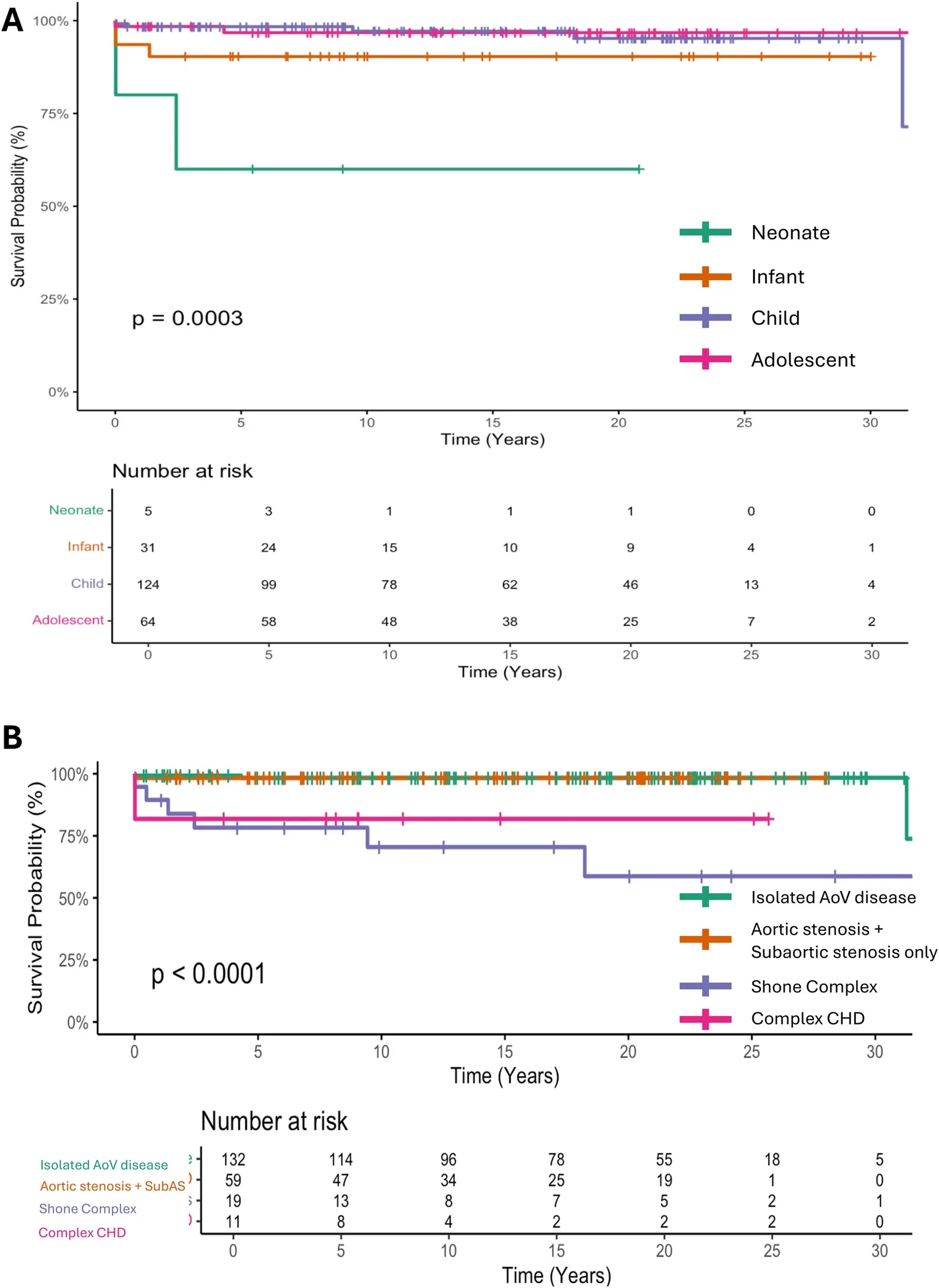
Kaplan-Meier survival curves following the Ross operation categorised by age group (A): neonates(≤28 days), infants (29 days-1 year), children(1-12 years), and adolescents (12-<18 years) and by diagnostic group (B): (1) isolated aortic valve disease; (2) aortic stenosis and subaortic stenosis only; (3) Shone complex; or (4) complex CHD (double outlet right ventricle, heterotaxy, hypoplastic aortic arch + ventricular septal defect or interrupted aortic arch).

Follow-up was complete in 96% of patients for a median duration of 9.5 ± 12.7 years (range: 0-33 years), with 210 (93%) patients alive at the time of data collection. In-hospital mortality was 2% (5 patients) with 3% (7 patients) late mortality. Median length of hospital stay was 7 ± 3 days and morbidity was seen in 29 patients (13%). When categorised by age group survival was lowest in the neonatal group, declining to 60% by 5 years with older groups demonstrating long-term survival above 90% at 30 years (Figure 1A). When categorised by diagnostic group, 25-year survival in the isolated aortic valve disease and aortic stenosis + subvalvar stenosis groups was 98.3% (95% CI: 95.1-100). In the complex CHD group, there was an initial hazard phase within the first-year post Ross but survival remained at 81.8% (95% CI: 61.9-100) thereafter. At 10 years the Shone complex group survival was 70.5% (95% CI: 51.3-96.8) and by 25 years this had decreased to 58.7% (95% CI: 36.4-94.7) (Figure 1B). When adjusted for age, diagnostic group was significantly associated with survival (*P* < .001). Compared to patients with isolated AV disease, those with Shone’s complex had an approximately 11-fold increased hazard risk of death (HR 11.4, 95% CI 2.4-54.6, *P* = .002), while those with complex CHD had an 8.8-fold increased hazard (HR 8.8, 95% CI 1.3-59.0, *P* = .025). Aortic stenosis + subvalvar stenosis was not associated with significantly different hazard (HR 0.72, 95% CI 0.07-7.34, *P* = .78).

### Fate of the autograft

**Figure 2.**
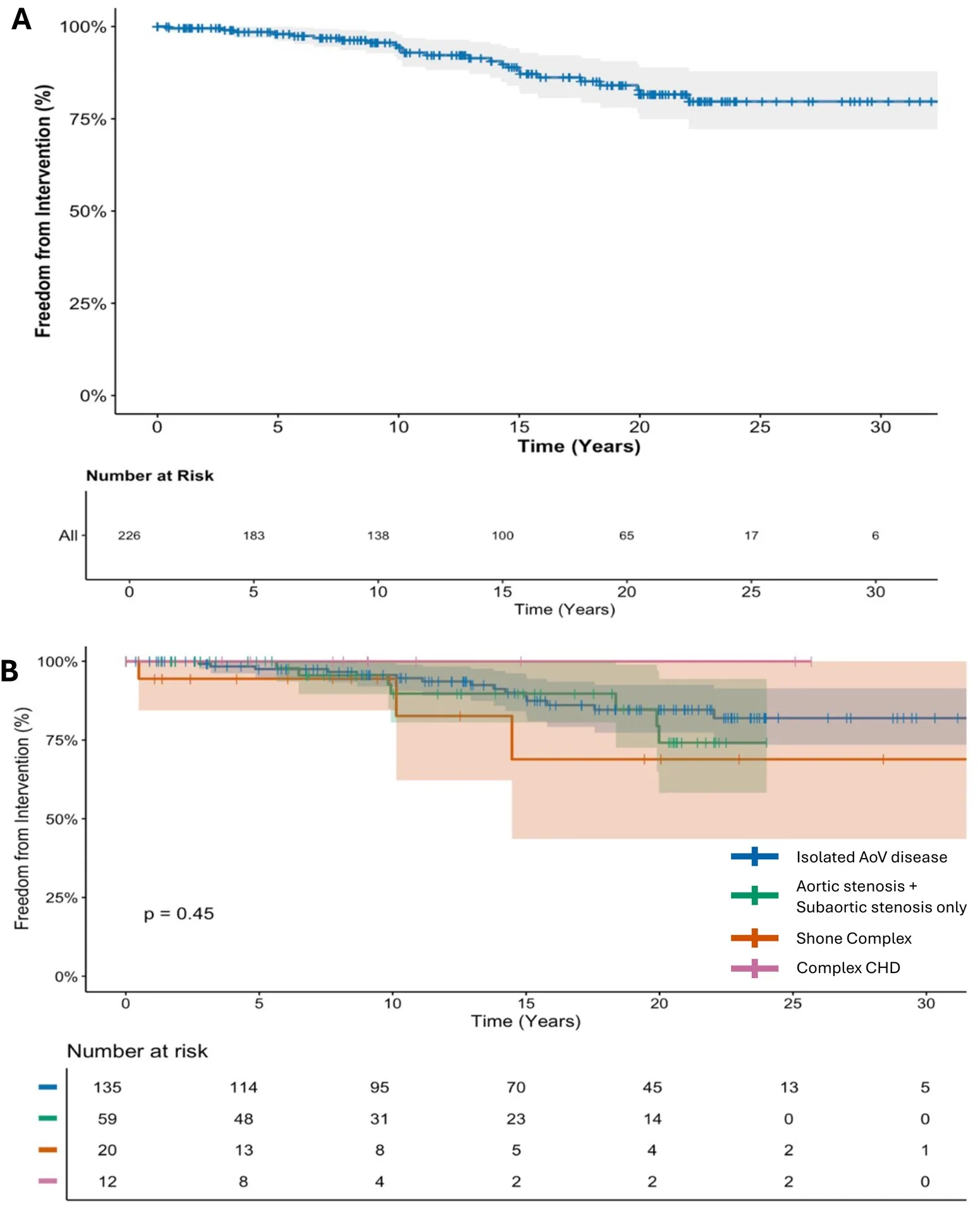
Overall freedom from autograft intervention following the Ross operation (A). Freedom from autograft intervention by diagnostic group (B).

A total of 25 (11%) autograft reinterventions were required, with an overall freedom from reintervention of 82% at 25 years (95% CI 75-90) (Figure 2A). There was no significant difference in freedom from autograft reintervention between the age groups (*P* = .85) or the diagnostic groups (*P* = .45). Similarly, on multivariable analysis, neither age nor diagnostic group was associated with autograft reintervention (Figure 2B). Patients were further stratified according to valve pathology and prior intervention status at time of Ross operation. When grouped into stenosis, native aortic regurgitation/mixed disease, and postintervention aortic regurgitation/mixed disease, there was no significant association with freedom from autograft reintervention (*P* = .9). Similarly, on multivariable analysis, valve pathology subtype was not predictive of autograft reintervention.

The most common indication for surgery was severe autograft dilatation (>50 mm) + severe aortic valve regurgitation [AR] (*n* = 15, 60%) followed by isolated severe AR (*n* = 5, 20%). Mechanical aortic root replacement was performed in 12 (48%) followed by valve-sparring root replacement (*n* = 4, 16%), mechanical aortic valve replacement (*n* = 4, 16%), and aortic valve repair (*n* = 4, 16%). Nineteen (76%) patients had at least 1 MRI scan prior to surgical reintervention. Of the 168 patients with complete echocardiographic data at discharge and latest follow-up, 68% had none or mild AR, 15% moderate AR, and 13% severe AR or had an autograft intervention. Patients with >mild AR at the time of discharge had a 17-fold risk of requiring autograft intervention.

**Figure 3.**
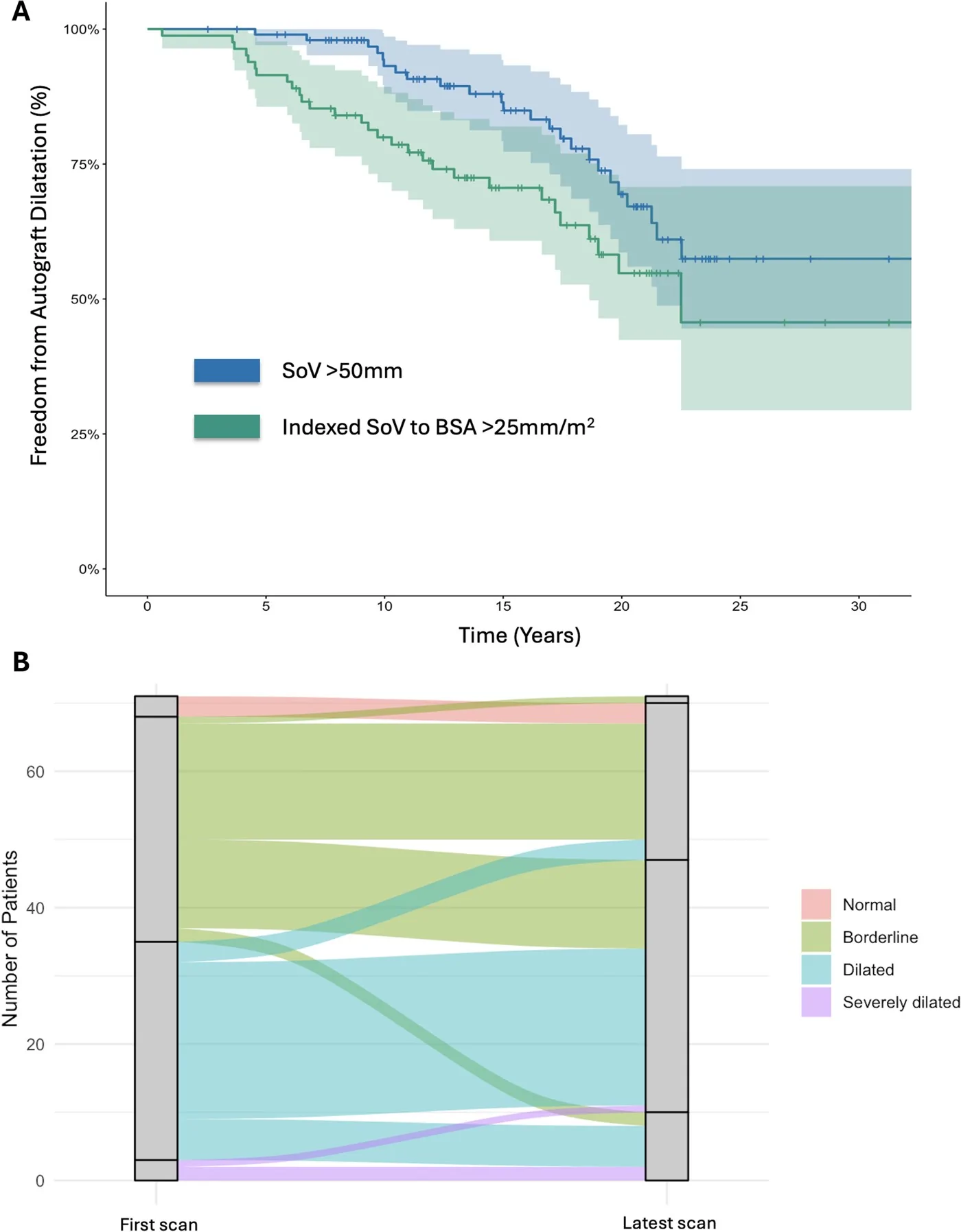
(A) Freedom from severe autograft dilatation in absolute sinus measurements (>50 mm) and sinus measurements indexed to body surface area—BSA (>25 mm/m^2^) obtained from follow-up MRI data. (B) Progression of autograft growth indexed to body surface area (BSA) between serial MRI scans during follow-up post Ross operation.^8^ Normal <20 mm/m^2^, dilated 20-25 mm/m^2^, severely dilated 25 mm/m^2^ (SoV—sinus of valsalva).

Among the overall cohort, 105 patients (46%) underwent at least 1 MRI scan post Ross, of whom 75 (33%) had multiple scans. The remaining 121 patients (54%) did not have MRI available. Within this subgroup, 23 patients (19%) required reintervention, including 6 patients who underwent autograft reintervention of which 3 required aortic root replacement. In contrast, patients undergoing MRI surveillance had a higher rate of autograft reintervention (17%), reflecting the selective use of MRI in patients with concerning clinical or echocardiographic findings. The median interval between the Ross procedure and the first MRI scan was 6.8 ± 6.1 years, while the median interval between the first and most recent MRI scan was 8.8 ± 5.5 years. The median autograft sinus growth during this time was 3.75 mm. Freedom from severe autograft dilatation (>50 mm) at 20 years was 70.8% (95% CI 60.3-83.1) and when indexed to BSA (>25 mm/m^2^) this was 54% (95% CI 28.8-69.9) at 20 years (Figure 3A).

In the 66 patients with digitally available complete BSA data (height/weight), 9 (14%) had a normal autograft size (<20 mm/m^2^) at the first MRI scan. Of these, 4 (44%) remained normal at follow-up, whereas 5 (56%) progressed to dilated (20-25 mm/m^2^). Thirty-seven patients (56%) were initially classified as dilated; 22 (59%) remained in the same category, 8 (22%) regressed to normal, and 7 (19%) progressed to severely dilated (>25 mm/m^2^). Among the 20 patients (30%) with a severely dilated autograft at baseline, 15 (75%) remained severely dilated and 5 (25%) improved to the dilated category (Figure 3B). Having a surgical intervention prior to the Ross operation did not reduce the hazard of developing severe aortic root dilatation (HR 0.8, 95% CI 0.36-1.8) or need for aortic valve reintervention (HR 0.6, 95% CI 0.28-1.3) *P* > .5.

### Fate of the RV-PA conduit

**Figure 4.**
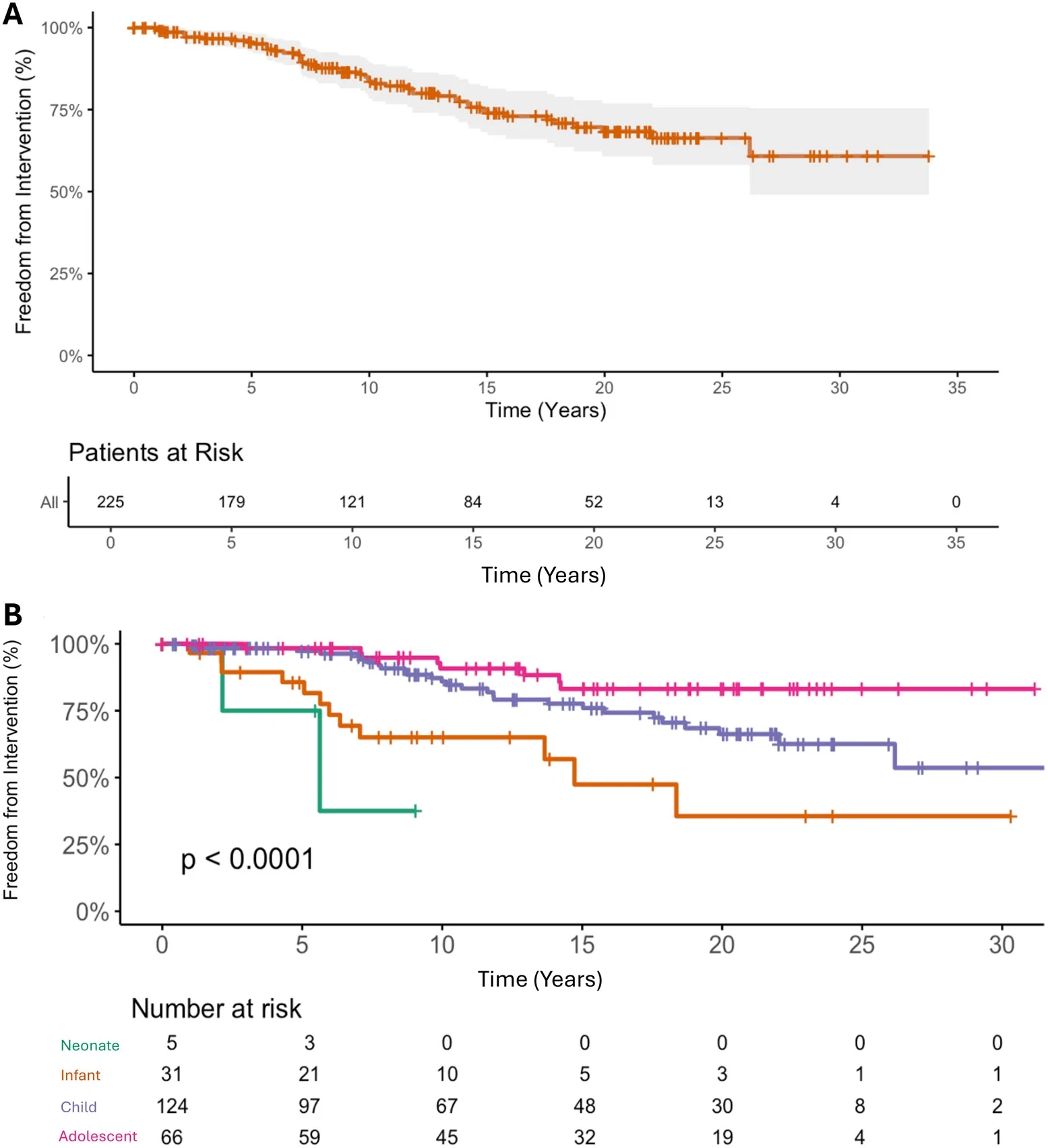
Overall freedom from intervention on the right ventricle to pulmonary artery (RV-PA) conduit following the Ross operation (A) and by age group (B).

Forty-five (20%) patients required surgery on the RV-PA conduit during follow-up. Overall freedom from reintervention was 66% at 25 years (95% CI 58.1-75.7) (Figure 4A). There were significant differences between the age groups (*P* < .001) with freedom from reintervention being lowest in neonates at <40% by 10 years. In the older cohorts 25-year freedom from reintervention was 36% (95% CI 16-77) in infants, 63% (95% CI 51-77) in children, and 83% (95% CI 73-95) in adolescents (Figure 4B).

## DISCUSSION

The Ross operation offers a durable, high-quality aortic valve replacement that closely resembles normal aortic valve haemodynamic function with physiological laminar flow contributing to consistently stable mean gradients over time.^10^^,^^11^ In the paediatric population further benefits include the growth capability of the autograft, the avoidance of anticoagulation and the suitability for smaller patients where there are no prosthetic substitutes available.^7^

This large, single-centre study assessed the long-term outcomes following the Ross operation in children and adolescents across more than three decades with particular focus on (i) overall survival, (ii) the fate of the autograft over time, and (iii) the impact of age at surgery and diagnostic category on long-term outcomes.

Operative mortality was low (2%) and overall mortality was 5%, which is consistent with other contemporary large series reporting excellent survival after the Ross operation in paediatric cohorts.^5^^,^^6^ Long-term survival exceeded 90% in patients older than 1 month, but was 60% in neonates, reflecting the complexity and comorbidities of this group. By diagnostic category, survival was 98% at 25 years in isolated aortic valve disease and aortic stenosis with subaortic stenosis. Patients with complex CHD experienced an early attrition, but survival then stabilised at 82%, while those with Shone complex had poorer outcomes with survival falling to 59% at 25 years with an 11-fold increased hazard of death. This likely reflects the diffuse and progressive nature of multilevel left heart obstruction associated with Shone complex. Unlike isolated aortic valve disease, these patients frequently have associated mitral valve abnormalities, subaortic obstruction, and recurrent left ventricular outflow tract disease, often requiring multiple interventions over time. This cumulative burden of disease may contribute to ventricular dysfunction and adverse long-term outcomes. These findings further emphasise that both age and underlying pathology remain critical determinants of outcome.^6^

### Autograft outcomes

Freedom from autograft reintervention was 82% at 25 years, comparable to previous reports in both paediatric and adult cohorts.^5^^,^^6^^,^^12^ The principal indication for reoperation was severe autograft dilatation with severe AR. In the absence of severe AR, we would elect to carefully monitor these patients, unless they required a reintervention on their RV-PA conduit, however the long-term outcome of these autografts remains unknown. Patients discharged at the time of Ross with >mild AR had a 17-fold greater risk of later autograft intervention, highlighting the importance of autograft valve competence at the index operation.

The impact of valve pathology, particularly the presence and mechanism of aortic regurgitation, has been proposed as a risk factor for autograft failure.^13^ In this study, stratification according to native versus postintervention aortic regurgitation did not demonstrate a significant association with autograft reintervention. These findings suggest that, within this cohort, preoperative valve phenotype was not a dominant determinant of autograft outcomes. In contrast, early post-operative autograft competence, as reflected by the presence of aortic regurgitation at discharge, appears to be a much stronger predictor of long-term failure.

Serial MRI demonstrated progressive sinus dilatation over time, with freedom from severe dilatation (>50 mm) of 71% at 20 years, and 54% when indexed to BSA. Although death is a competing risk for this end-point, the number of deaths in the cohort was small (*n* = 12), and the impact on the reported estimates is therefore minimal.

In the subset with serial imaging, most patients remained in their initial category, though some showed regression or progression. These observations are consistent with prior studies demonstrating autograft remodelling over time.^5^^,^^7^^,^^11^^,^^14–16^ Collectively, the literature highlights that while the Ross confers physiological advantages, autograft dilatation remains an important long-term concern, particularly in the presence of AR.^5^^,^^11^ Preventive strategies such as subcoronary implantation, root inclusion, annular reduction, or external reinforcement have shown promise in mitigating dilatation in both paediatric and adult series, although it remains unclear whether this improves overall autograft durability.^11^^,^^17^^,^^18^ Our findings reinforce the need for lifelong surveillance and raise important questions about thresholds for intervention in patients with significant root dilatation but preserved valve function. MRI normative data suggest an upper normal limit around 21-23 mm/m^2^ for the adult aortic root indexed to BSA; accordingly, we defined severe dilation as >25 mm/m^2^, corresponding to ∼3-4 standard deviations above the mean in healthy cohorts, although this threshold remains less well defined in paediatric and young adult populations.^8^ In paediatric populations, aortic dimensions are typically indexed to body surface area. In adults, however, there has been a shift towards indexing to height, which provides a more consistent measure, demonstrates comparable predictive value to BSA, and avoids variability introduced by weight.^19^ As paediatric Ross patients transition into adulthood during follow-up, height-based indexing may be a reasonable approach, although this was not applied in the present study.

In addition, autograft root dilatation and aortic regurgitation appeared to be closely related. The most common indication for reintervention was the combination of significant root dilatation and severe aortic regurgitation, suggesting that progressive root enlargement may contribute to loss of valve coaptation. Conversely, aortic regurgitation may increase volume load and promote further remodelling. However, the relationship between these processes could not be fully defined due to the retrospective study design and incomplete longitudinal imaging.

### Impact of prior interventions and timing of the Ross

In our cohort, prior surgical or catheter-based interventions did not protect against autograft dilatation or later reintervention. These findings argue against delaying the Ross in favour of interim procedures, consistent with previous studies that concluded that earlier Ross implantation better preserves LV function and reduces the cumulative burden of intervention.^7^^,^^11^^,^^20^ Histological studies have shown that autografts may have greater adaptive potential in the first 2 years of life.^21^ This could be supported by lower systemic blood pressures, smaller stroke volumes, which reduce the haemodynamic stress on the autograft potentially delaying dilatation or regurgitation. Previous reports suggest that early implantation in infants can be safe, provided it is performed in specialist centres.^7^

### Neonatal Ross

The neonatal cohort showed the highest mortality and conduit reintervention burden. These patients often represent the sickest subgroup with no alternative surgical options, and outcomes are inferior compared with those in older children. Previous studies including meta-analyses have reported early mortality of 12%-38%.^5^^,^^22^ Our results are consistent with these findings. However, it is important to note that mortality is markedly lower in patients >28 days, highlighting that the Ross should be considered a safe and effective operation outside of the neonatal setting.

### RV-PA conduit outcomes

Reintervention on the RV-PA conduit was required in 20% of patients, with freedom from reintervention of 66% at 25 years. As expected, reintervention rates were highest in neonates and infants, largely reflecting somatic outgrowth, with outcomes progressively improving in older children and adolescents. This pattern is consistent with previous reports.^5^^,^^6^^,^^11^ While conduit choice in our series was predominantly pulmonary homograft, alternative conduits were too few to allow meaningful comparisons, although previous studies have demonstrated better durability in pulmonary and aortic homografts.^23^

Based on over three decades of experience, several key principles emerge. Careful patient selection is critical, with the most favourable outcomes in those with isolated aortic valve disease ± subvalvar stenosis. Early autograft competence is paramount, as >mild residual aortic regurgitation is strongly associated with late autograft failure. Outcomes remain less favourable in neonates and in patients with multilevel left heart disease, particularly Shone’s complex. Lifelong surveillance is essential given the ongoing risk of autograft dilatation and RV-PA conduit failure.

### Limitations

This study is limited by its retrospective design and single-centre nature, which limits the generalisability of these findings. However, across the 3 decades with 6 surgeons, outcomes remained consistent. Not all patients underwent serial cross-sectional imaging, and MRI surveillance was not performed systematically but was guided by clinical indication. In practice, patients with evidence of autograft dilatation or aortic regurgitation on echocardiography were more likely to undergo MRI assessment, introducing selection bias. This approach is consistent with contemporary series in which cross-sectional imaging is performed selectively rather than routinely.^9^ As a result, the MRI cohort likely represents a higher-risk subgroup, and findings related to autograft dimensional progression should be interpreted accordingly. Small subgroup sizes, particularly in neonates and Shone complex, reduce statistical power. Nevertheless, the large overall cohort, completeness of follow-up, and detailed imaging and surgical data strengthen the validity of these findings.

## CONCLUSION

The Ross operation provides excellent long-term survival and autograft durability in children and adolescents, particularly those with isolated aortic valve disease or associated subaortic stenosis. While autograft dilatation and RV-PA conduit reintervention remain important considerations, overall freedom from reintervention is high. Early post-operative AR strongly predicts later autograft failure, emphasising the importance of valve competence at the index operation. These findings support the Ross as the preferred surgical option for young patients with aortic valve disease, provided lifelong surveillance in specialised centres.

## Data Availability

The data underlying this article will be shared on reasonable request to the corresponding author.

## Abbreviations

AR: aortic regurgitation
AV: aortic valve
BSA: body surface area
CHD: complex congenital heart disease
ECMO: extracorporeal membrane oxygenation
HR: hazard ratio
IQR: interquartile range
LV: left ventricle
LVOTO: left ventricle outflow obstruction
MRI: magnetic resonance imaging
RV-PA: right ventricle-pulmonary artery
STJ: sinotubular junction
